# Corrigendum to “TDP-43-M323K causes abnormal brain development and progressive cognitive and motor deficits associated with mislocalised and increased levels of TDP-43” [Neurobiology of disease Volume 193, April 2024, 106437]

**DOI:** 10.1016/j.nbd.2024.106708

**Published:** 2024-11

**Authors:** Juan M. Godoy-Corchuelo, Zeinab Ali, Jose M. Brito Armas, Aurea B. Martins-Bach, Irene García-Toledo, Luis C. Fernandez-Beltrán, Juan I. Lopez-Carbonero, Pablo Bascunana, Shoshana Spring, Irene Jimenez-Coca, Ramón A. Muñozde Bustillo Alfaro, Maria J. Sanchez-Barrena, Remya R. Nair, Brian J. Nieman, Jason P. Lerch, Karla L. Miller, Hande P. Ozdinler, Elizabeth M.C. Fisher, Thomas J. Cunningham, Abraham Acevedo-Arozena, Silvia Corrochano

**Affiliations:** a Neurological Disorders Group, Hospital Clínico San Carlos, Instituto de Investigación Sanitaria Hospital Clínico San Carlos (IdiSSC), Madrid 28040, Spain; b MRC Harwell Institute, Oxfordshire, UK; c Unidad de Investigación, Hospital Universitario de Canarias, ITB-ULL and CIBERNED, La Laguna, Spain; d Wellcome Centre for Integrative Neuroimaging, University of Oxford, Oxford, UK; e Department of Medicine, Universidad Complutense de Madrid, Madrid, Spain; f Brain Mapping Group, Hospital Clínico San Carlos, IdISSC, Madrid, Spain; g Mouse Imaging Centre, The Hospital for Sick Children, Toronto, ON, Canada; h Department of Crystallography and Structural Biology, Institute of Physical Chemistry “Blas Cabrera”, CSIC, Madrid, Spain; i Nucleic Acid Therapy Accelerator (NATA), Harwell Campus, Oxfordshire, UK; j Department of Neurology, Feinberg School of Medicine, Northwestern University, Chicago, IL, USA; k Department of Neuromuscular Diseases, UCL Queen Square Motor Neuron Disease Centre, UCL, Institute of Neurology, London, UK; l MRC Prion Unit at UCL, UCL Institute of Prion Diseases, London, UK

The authors regret that acknowledgment to a funder is missing in article YNBDI_106437.

RMBA is co-funded by 10.13039/501100007757ACIISI of the Consejería de Economía, Industria, Comercio y Conocimiento and 10.13039/501100004895Fondo Social Europeo.

The authors would like to apologise for any inconvenience caused.

