# Supplementary material for: TDP-43-M323K causes abnormal brain development and progressive cognitive and motor deficits associated with mislocalised and increased levels of TDP-43
Source: Neurobiol Dis. 2024 Apr;193:106437. doi: 10.1016/j.nbd.2024.106437 (PMC10988218; doi:10.1016/j.nbd.2024.106437)
Supplement: Supplementary file 1 — Supplementary material: Supplementary Figures 1-5 [file mmc1.docx]

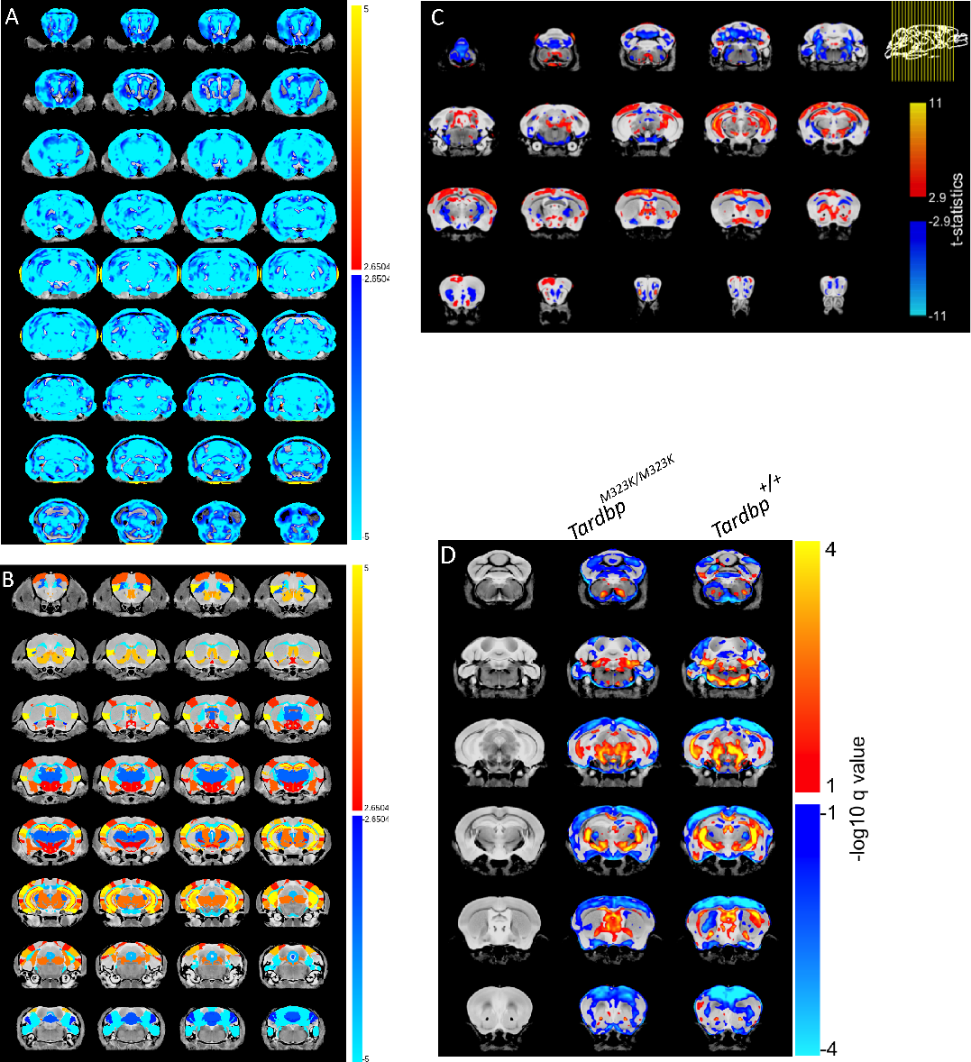


**Supplementary Figure 1.**

**A.** Decrease in total volume in the whole brain at 12 months of age. **B.** Relative changes in different areas of the brain at 3 months of age. **C.** Relative changes in different areas of the whole brain at 12 months of age. **D.** Changes in different areas of the brain due to the age in wild-type and homozygous mice. (At 3 months, n = 6-7 per group, at 12 months, n = 8 per group).


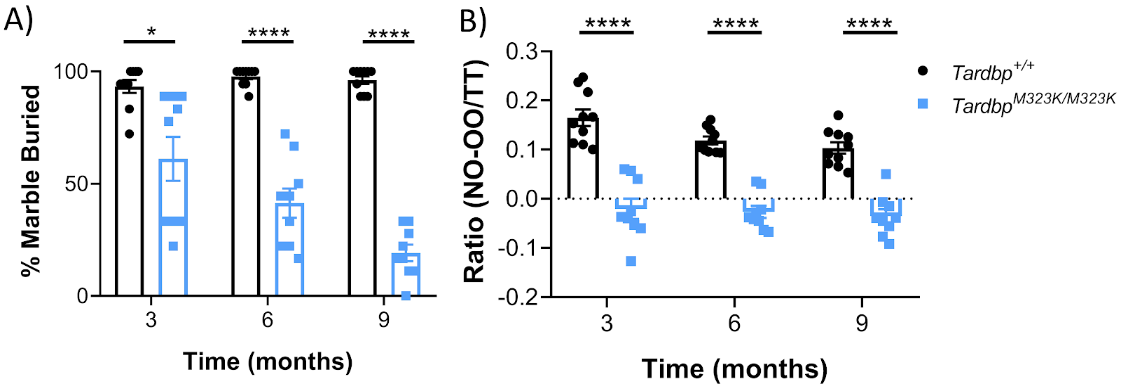


**Supplementary Figure 2**

**A.** Marble burying test. The percentage of marbles two-thirds buried was recorded. (n = 9-10 mice per group). **B.** NOR test. To quantify the NOR result, we use a ratio that is calculated as follows: (time on the new object minus time on the known object) divided by the total test time (300 sec) (n = 9-10 mice per group). Data were analysed using two-way ANOVA. Data in graphs represent the mean±S.E.M. *P<0.05, ****P<0.0001.

**
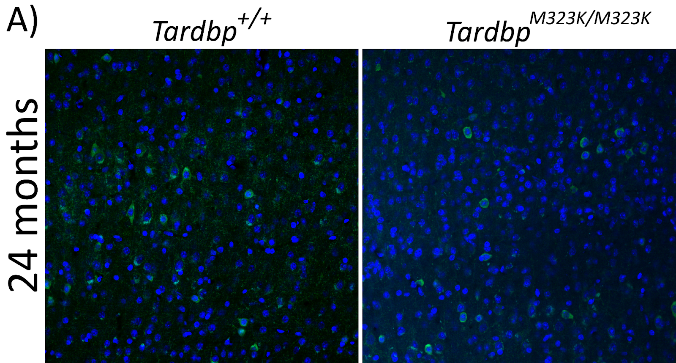
**

**Supplementary Figure 3**

**A.** Representative confocal images of the frontal cortex from female mice at 24 months of age. Merged with Parvalbumin staining (in green) and nuclei (stained with DAPI, in blue).

**
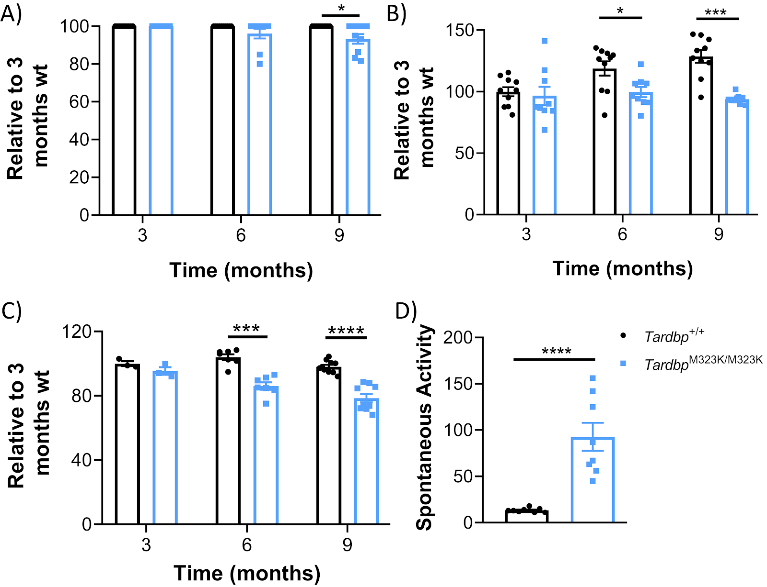
**

**Supplementary Figure 4**

**A.** Grid test. Grid test in *Tardbp^+/+^* and *Tardbp^M323K/M323K^* male mice at 3, 6 and 9 months of age (n = 9-10 per group). **B.** Grip strength test. Corrected means for individual animals’ body weight normalized to 3 months *Tardbp^+/+^* (n = 9-10 per group). **C.** CMAP amplitude in the hind limbs at 3, 6 and 9 months of age. **D.** Spontaneous activity at 9 months of age. Presence of spontaneous activity in *Tardbp^M323K/M323K^* (n = 9-10 per group). *P<0.05, ***P<0.001, ****P<0.0001.

**
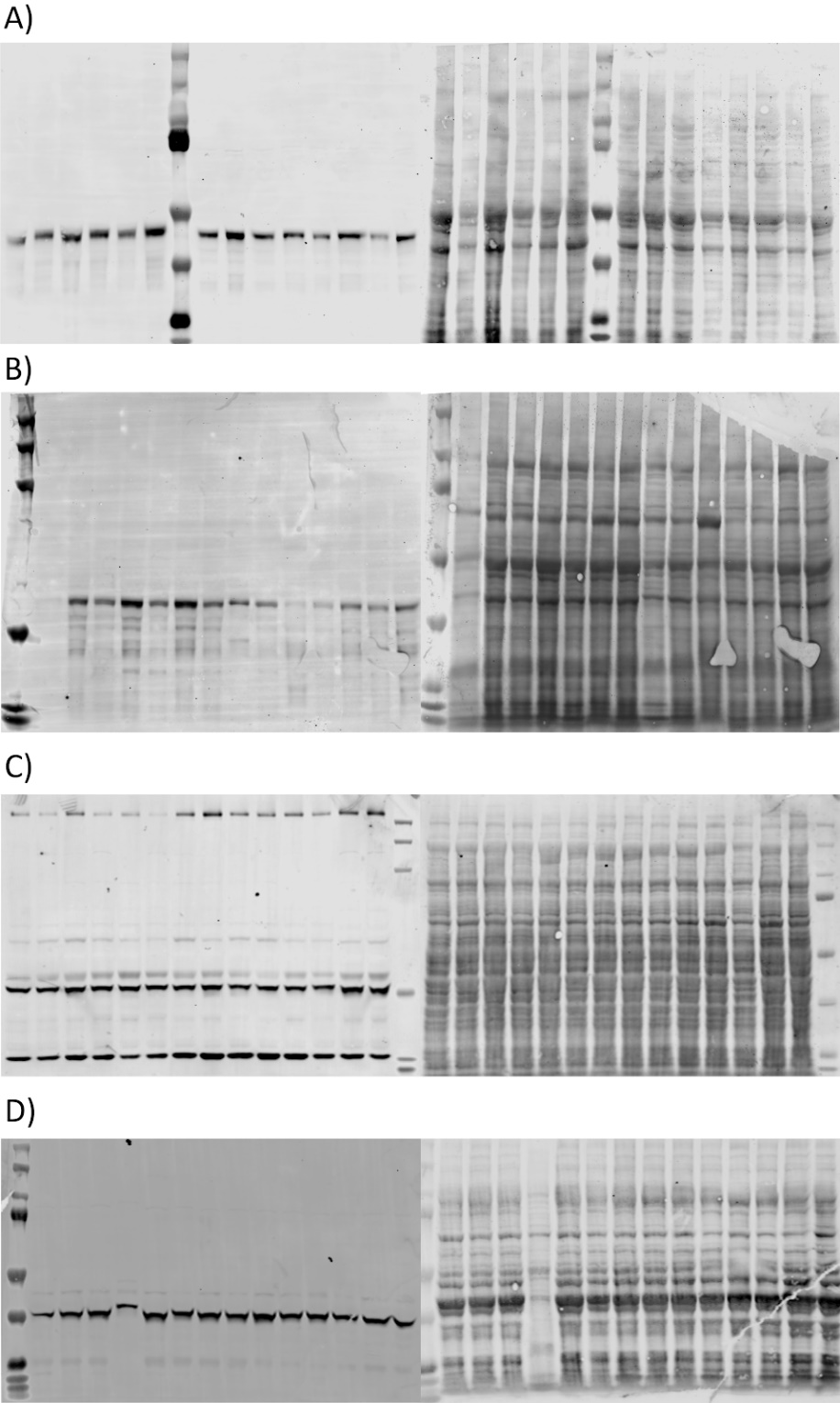
**

**Supplementary Figure 5**

An immunoblot with dilution of 1:1,000 of TDP-43 antibody (ThermoFisher, #MA532627) **A.** Frontal Cortex, **B.** Spinal Cord, **C.** Liver, **D.** Tibialis anterior (n = 3-4 per group and time point).
